# Dental Approach to Craniofacial Syndromes: How Can Developmental Fields Show Us a New Way to Understand Pathogenesis?

**DOI:** 10.1155/2012/145749

**Published:** 2012-10-02

**Authors:** Inger Kjær

**Affiliations:** Department of Orthodontics, Institute of Odontology, Faculty of Health Sciences, University of Copenhagen, 20 Nørre Allé, DK-2200 Copenhagen, Denmark

## Abstract

The paper consists of three parts. *Part 1: Definition of Syndromes*. Focus is given to craniofacial syndromes in which abnormal traits in the dentition are associated symptoms. In the last decade, research has concentrated on phenotype, genotype, growth, development, function, and treatment. *Part 2: Syndromes before Birth*. How can the initial malformation sites in these syndromes be studied and what can we learn from it? In this section, deviations observed in syndromes prenatally will be highlighted and compared to the normal human embryological craniofacial development. Specific focus will be given to developmental fields studied on animal tissue and transferred to human cranial development. *Part 3: Developmental Fields Affected in Two Craniofacial Syndromes*. Analysis of primary and permanent dentitions can determine whether a syndrome affects a single craniofacial field or several fields. This distinction is essential for insight into craniofacial syndromes. The dentition, thus, becomes central in diagnostics and evaluation of the pathogenesis. Developmental fields can explore and advance the concept of dental approaches to craniofacial syndromes. *Discussion*. As deviations in teeth persist and do not reorganize during growth and development, the dentition is considered useful for distinguishing between syndrome pathogenesis manifested in a single developmental field and in several fields.

## 1. Introduction

Syndrome research reveals detailed insight into different aspects of congenital malformation of several body components. These malformations affect physical and psychological postnatal development and treatment. Such a wide-spread field of interest requires multidisciplinary teamwork, including clinicians and researchers with different backgrounds. In the last decade, focus has specifically been given to pathogenesis and genotype. A complete overview of craniofacial syndromes would involve not only the dental approach, but also several medical, psychological, and neurological clinical and theoretical approaches. The present paper focuses on 3 key aspects in craniofacial syndromes. These are definition and classification of dental deviations (Part 1); appearance of malformations before birth (Part 2) and a specific new dental approach to craniofacial syndromes (Part 3).

## 2. Part 1: Definition of Syndromes

Spranger et al. [1] defined syndromes accordingly: “A syndrome is a pattern of multiple anomalies thought to be pathogenetically related and not known to represent a single sequence or a polytopic field defect”. Furthermore, Spranger et al. defines “sequence” as “A pattern of multiple anomalies derived from a single known or presumed prior anomaly or mechanical factor”. A polytopic field defect is defined as “A pattern of anomalies derived from the disturbance of a single developmental field” [1].

Dorland's Illustrated Medical Dictionary defines syndrome as “A set of symptoms that occur together; the sum of signs of any morbid state; a symptom complex. In genetics, a pattern of multiple malformations thought to be pathogenetically related” [2]. 

A craniofacial syndrome is characterized by morphological and developmental deviations in the cranial tissue components, including teeth. It is difficult to determine whether the deviation has developed at a primary location that subsequently causes secondary alterations or whether there are general deviations in many structures and at many locations.

In some craniofacial syndromes, the genotype is known and in some it is unknown. Even though the genotype is known in a craniofacial syndrome, it is not understood why that particular genotype causes the malformations observed. A known genotype deviation can manifest itself in the cranium by many different phenotypic expressions, from severe to minor affection. How and why the genotype affects the cranial development and why it affects the cranial components differently are far from explained in full.

In the foreword to the textbook *Syndromes of the Head and Neck* by Gorlin et al. [3] F. Clarke Fraser stated that the symptoms in craniofacial syndromes may not be limited to the head and neck. It can therefore be questioned whether there are craniofacial syndromes that are limited to the head and neck. This is a challenge for future research.

In this section, focus is given to studies on craniofacial syndromes in which abnormal traits in the dentition are associated symptoms. Such abnormal traits could be deviations in tooth number (agenesis, supernumerarity), tooth morphology (size, dimensions, crown invaginations, and abnormal shapes of crowns and roots), tooth eruption (delayed eruption, ankylosis), and resorption (crowns and roots). Occurrence of these dental deviations is classified and exemplified in the following.

### 2.1. Agenesis

Agenesis has been reported in several syndromes. Schalk-van der Weide [4] reported that specific patterns of agenesis was associated with specific syndromes. Thus, absence of the following teeth was observed in these syndromes: Böök's syndrome: premolars; Rieger syndrome: maxillary incisors (constant), mandibular incisors, and premolars (occasional); Ellis-van Creveld syndrome: mandibular incisors and canines; Gorlin-Chaudry-Moss syndrome: deciduous molars, secondary premolars, and molars; Lipoid proteinosis: maxillary lateral incisors, canines, and premolars; Coffin-Lowry syndrome: maxillary lateral incisors, mandibular incisors; Orofaciodigital syndrome Type I: mandibular lateral incisors and canines; Down syndrome: third molars, mandibular second premolars, and maxillary lateral incisors; Holoprosencephaly: maxillary incisors; Hypoglossia-hypodactylia syndrome: mandibular incisors and canines; Glossopalatine ankylosis syndrome: incisors.

### 2.2. Supernumerary Teeth

Supernumerary teeth have been reported in Cleidocranial dysplasia [9].

### 2.3. Tooth Morphology

With regards to tooth morphology, large teeth have been reported in KBG syndrome where macrodontic incisors have been described [10]. Microdontic teeth have been reported in Down syndrome [11]. Deviations in tooth dimensions such as short roots and malformations of crowns and roots have been observed in Turner syndrome [12], in Klinefelter syndrome [13], and in Rothmund-Thomson syndrome [14].

### 2.4. Eruption

Delay in eruption has been reported in Down syndrome [15]. Complete failure of eruption has been observed in Gapo syndrome [16]. Other eruption deficiencies have also been observed.

### 2.5. Resorption

Delayed resorption of primary teeth has been observed in Cleidocranial dysplasia [9] and in Hyper IgE syndrome [17].

These symptoms of deviations in the dentition are all phenotypic traits of the specific syndromes mentioned. Why it is so is still not known.

Craniofacial research is currently focused especially on determination of genotypes of different syndromes [6, 18–21].

Research is also devoted to phenotypic descriptions of craniofacial syndromes [22–24].

From a treatment point of view, interest has specifically been given to the use of dental implants. Yap and Klineberg [25] stated that the success rate is lower for implants in patients with ectodermal dysplasia than it is in patients with tooth agenesis but without ectodermal dysplasia.

Optimal treatment in multidisciplinary teams is also given some focus (e.g., [26]).

In the central textbook on craniofacial syndromes [3], Gorlin et al. stated that one-third of children born with birth defects display anomalies in the head and neck. He also reported that 72 different syndromes with orofacial cleft were registered in 1971 and that this number had increased to 250 syndromes in 1990. This development involves all craniofacial phenotypes and new findings document that craniofacial phenotypes are gradually associated with symptoms outside the craniofacial area. In 2010, Trainor [27] reported that more than 700 craniofacial malformations have been described.

The goal for future research on craniofacial syndromes must be to understand the connection between symptoms in the dentition and other symptoms in the craniofacial area.

## 3. Part 2: Syndromes before Birth

How can the initial malformation sites in these syndromes be studied and what can we learn from it? Prenatal syndromes can be studied by ultrasound or by autopsy after spontaneous or provoked abortion.

Research on human prenatal autopsy material for demonstration of craniofacial development has a morphological character and is predominantly based on histological, histochemical, and radiographic studies. These studies depend entirely on autopsy legislation in different countries and represent primarily the embryonic period and the foetal period before the 16th week of gestation.

In contrast to human studies, experimental studies on the craniofacial development in animals allow for a variety of advanced procedures, such as cell cultures and labelling of migrating cells, and may cover the complete foetal period [28, 29].

In a series of human autopsy studies, the initial sites of malformation in craniofacial syndromes have been investigated. Of specific interest are studies on Holoprosencephaly [30, 31], Anencephaly [32–35], amniotic band rupture [36], Down syndrome (Trisomy 21) [37–40], other trisomies [41–45], Meckel syndrome [46, 47], orofacial clefts [48–50], and cranial encephalocele [51].

In order to evaluate these pathologies, it is necessary to know the initial sites of normal craniofacial development before birth. Normal development has been described in several studies and comprised in the textbook *The Prenatal Human Cranium* [52].

When normal development is compared with the pathologic conditions mentioned above, the following is observed.

### 3.1. Holoprosencephaly

The deviation is located in a midsagittal field between the eyes and spans backwards to the sella turcica. This field is called the frontonasal field.

### 3.2. Anencephaly

The cranial deviation corresponds to the desmal-formed theka crania, but the cartilaginous originated part of the occipital squama is not malformed. Other cranial structures may be deformed due to the absent cerebrum, but the structures are present. It is interesting that the neural hypophysis is absent.

### 3.3. Amniotic Band Rupture

Though amniotic band rupture in the cranium appears as a craniofacial syndrome, it is not a congenital malformation and not a syndrome. Analyses of the condition reveal that rupture of a normal developmental course has occurred and caused extensive disruptions, not limited to developmental regions (fields).

### 3.4. Down Syndrome

The cranial phenotype in foetuses with Down syndrome is characterized by short or absent nasal bone, short and abnormal, often cleft, palate, deviations in size and dimensions of the occipital area (the occipital or cerebellar field), and malformations in the sella turcica. Cervical spine malformation is a constant finding. Thus, deviations occur in many regions of the cranium.

### 3.5. Other Trisomies

The craniofacial skeleton in Trisomy 18 and 13 and Triploidy has been studied and it was shown that the developmental deviations are completely different in these trisomies. This indicates an early and specific interrelationship between genotype and phenotype.

### 3.6. Meckel Syndrome

In Meckel syndrome, deviations are seen in the occipital region and sella turcica.

### 3.7. Orofacial Clefts

The nasal bone is short in cleft lip, but not in cleft palate and combined cleft lip and palate. The sella turcica deviates especially in combined cleft lip and palate. Abnormal findings were seen in the cranial base/sella turcica and the nasal bone/maxilla.

### 3.8. Cranial Encephalocele

Different malformations are observed in bones in different types of cranial encephaloceles. Interestingly, the same type of malformation occurs in the sella turcica/pituitary gland region in different types of encephalocele.

### 3.9. Preliminary Conclusion

A general conclusion from craniofacial studies in the foetal period is that an association exists between the development of the cranium and the body axis/cervical spine and between the cranium and the central and peripheral nervous systems (CNS/PNS). This is not surprising as studies on the early gastrulation in the embryonic period have shown an axial midline structure in the body, the primitive streak [53], which will later involve the notochord and the head formation. The rostral extent of the notochord appears in the posterior wall of the sella turcica [54].

Concerning cranial development, the main conclusion is that different regions of the cranium are malformed in different genotypes. These cranial regions are called fields. In order to understand these fields, we need to turn to experimental studies on animals. 

### 3.10. Developmental Fields

Experimental studies performed by Le Douarin et al. [28, 55] on animal tissue have revolutionized the embryological understanding of the craniofacial development. In these studies the neural crest cells from different specific regions were labelled and the authors discovered that cells from different regions on the neural tube migrate to different specific parts of the cranial face and dentition, thus forming different developmental fields.

**Figure 1:**
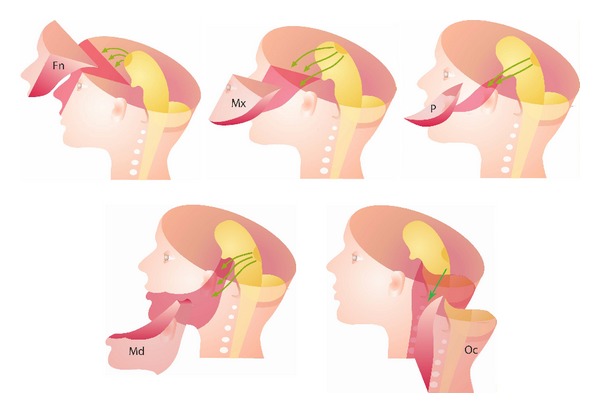
Schematic illustrations of craniofacial fields. Fn: frontonasal field, Mx: maxillary field, P: palatal field, Md: the complete mandibular field, and Oc: occipital and cervical spine field. Green arrows indicate migration paths of neural crest cells from different regions at the neural tube to different developmental fields in the cranium.

**Figure 2:**
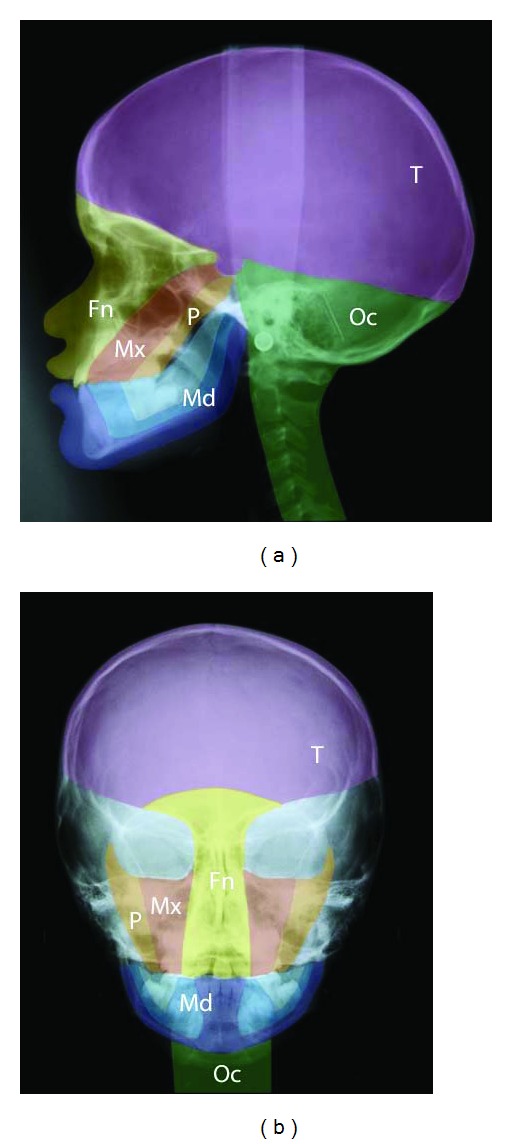
Profile (a) and frontal (b) radiograph of a girl aged 9 years with a single median maxillary central incisor. Different developmental fields are marked on the radiographs. Green: occipital and cervical spine field (Oc). Purple: theka field (T). Light and dark blue: the complete mandibular field (Md). Yellow: frontonasal field (Fn). Red: maxillary field (Mx). Orange: palatal field (P). Note that the sella turcica is a borderline region between fields. This figure is reprinted with permission from European Journal of Orthodontics 2010:32:140-147 [5].

**Figure 3:**
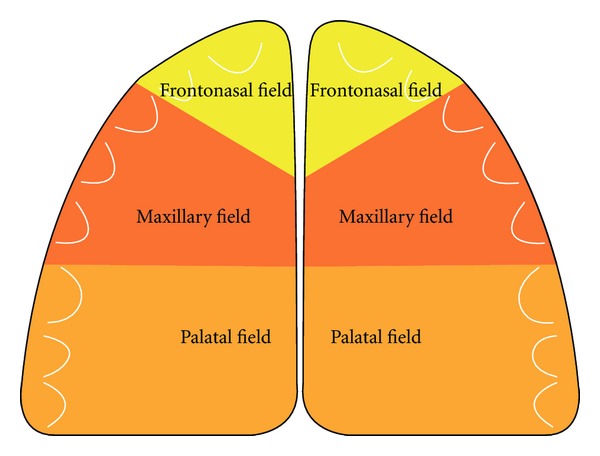
Schematic drawing of the maxilla, demonstrating three different fields in the left and right side of the maxilla.

**Figure 4:**
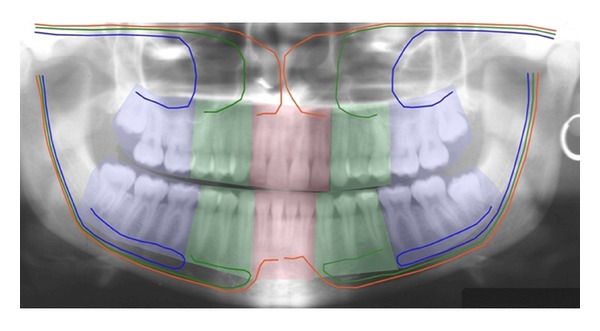
Panoramic radiograph illustrating the different fields in the maxilla and mandible with different innervation, surrounded by different ectomesenchyme. In the maxilla: red colour demonstrates the bilateral frontonasal field, innervated by the nervus nasopalatinus. Green colour demonstrates the bilateral maxillary field, innervated by the nervus maxillaris. Blue colour demonstrates the bilateral palatal fields, innervated by the nervus palatinus. In the mandible, similar fields are illustrated, innervated by different nerve branches from the nervus alveolaris inferior [6]. This figure is reprinted with permission from Orthodontic Waves 2012;71:1-16 [7].

In order to understand the human craniofacial development, it is necessary to combine morphological human studies with knowledge acquired through experimental studies regarding cell migration. Such combination studies have led to insight into developmental fields in humans [5, 7]. The developmental fields in the human cranium are illustrated in Figures 1 and 2 and in the dentition in Figures 3 and 4.

Spranger et al. [1] defined a developmental field as “A region or part of an embryo which responds as a coordinated unit to embryonic interaction and results in complex or multiple anatomical structures. An instrinsic, nondisruptive disturbance of a developmental field will lead to a field defect” [1]. The developmental fields in the maxilla described in the present paper are the frontonasal field, the maxillary field, and the palatal field. These three bilateral fields arise from different regions on the neural crest and have three different main nerve supplies: nervus nasopalatinus, nervus maxillaris, and nervus palatines, respectively [56]. Three similar developmental fields exist in the mandible, innervated by different nerve branches, which are connected during development and mandibular growth in the bundle of peripheral nerves named the inferior alveolar nerve [57]. The interrelationship between the central and peripheral nervous systems has been studied intensively in human foetal pathological studies [58].

**Figure 5:**
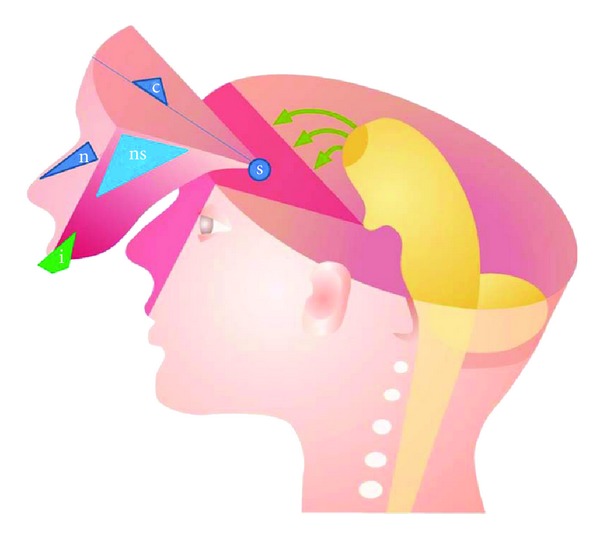
Schematic illustration of hard tissue structures within the frontonasal field in the human cranium. s: sella turcica; c: crista galli; ns: nasal septum; n: nasal bone; i: central incisor.

Developmental fields in the cranium extend in a triangular shape from the pituitary gland/sella turcica region to an outer region of the face. The sella turcica/pituitary gland region is the end region for the rostral extension of the notochord. It is therefore necessary, when describing a craniofacial developmental field, to describe the complete field in its 3-dimensional extent. The field that has been described most in detail based on foetal pathological observations is the frontonasal field [30, 34] (Figure 5). This field includes the anterior wall of the sella turcica, the anterior cranial base, the interocular region, nose, and philtrum (Figure 5). Within this field, the involvement of the vomeronasal organs producing luteinizing hormone-releasing hormone, LHRH [59], and the sella turcica/pituitary gland [60, 61] can often explain hormone-related deviations in body height and maturity associated with deviations in the frontonasal field [5].

It has been shown that syndromes, for example, Velocardiofacial syndrome located in the palatal field in the cranium, also involve organ structures such as the brain, thymus, thyroid, and heart septum [62]. It is not obvious that malformations of these organs are caused by the same field defect, but it may be explained from the neural crest cell migration in this region [62].

Another example of craniofacial malformations interrelated with organ malformations can be seen in Cri du Chat syndrome where the cerebellum and larynx, located far apart, are developmentally associated with the posterior cranial base [63].

## 4. Part 3: Developmental Fields Affected in Craniofacial Syndromes

Analysis of the primary and permanent dentitions can determine whether a syndrome affects a single craniofacial field or several fields. The previous section showed how prenatal studies are useful for analysis of the initial site of organ and osseous malformations, but due to the early period of development, less useful for analysis of the dentition. Only early traces in the development of the primary teeth can be analyzed prenatally. In contrast to the prenatal tissue analysis, postnatal analysis on radiographic material is highly useful for analysis of primary and permanent teeth, but not useful at all for analysis and limitation of developmental fields. A combination of prenatal and postnatal insight is therefore necessary in order to understand how developmental fields are affected in different craniofacial syndromes.

Opitz [64] has defined developmental fields as the morphogenetic units of the embryo. He also states that processes in developmental fields are self-organizing spatially coordinated and ordered, epimorphically hierarchical, temporarily synchronized, epigenetically interactive, developmentally constrained, and phylogenetically conserved [64].

### 4.1. Single Field, Exemplified by SMMCI

In a craniofacial syndrome where deviations occur in a specific field, it is expected that all structures, including teeth, within the field can be deviant. Structures outside the field are not necessarily deviant. In this section, focus will be on the SMMCI syndrome (single median maxillary central incisor) with gene location 7q36.3 [65]. In this syndrome, the frontonasal field is affected. The craniofacial examination of this condition reveals a highly significantly shorter anterior cranial base [66].

In SMMCI, only one maxillary central incisor exists in both the primary and permanent dentitions [67, 68]. The malformation has been associated with sonic hedgehog gene, which normally defines the midaxial part of the frontonasal field [31, 69]. As a result, the midaxial structures of the face, maxilla, nasal cavity, nasal bone, anterior cranial fossa, and sella turcica are malformed. The sella turcica is often tiny and malformed and the growth hormone production in the pituitary gland deviates [8]. Children with SMMCI are accordingly often short in stature.

**Figure 6:**
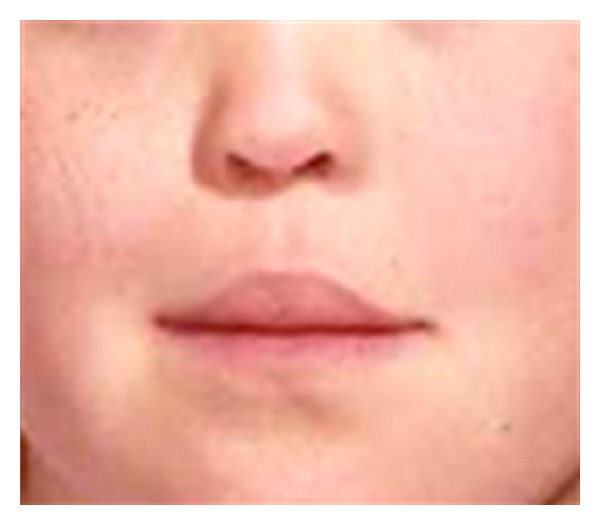
Section of photograph of a girl, aged 12 years 1 month, with SMMCI (single median maxillary central incisor). The face is characterized by tubular-shaped nose and philtrum blurred towards the prolabium without the normal s-shape. Deviations all occur within the frontonasal field, illustrated schematically in Figure 5.

**Figure 7:**
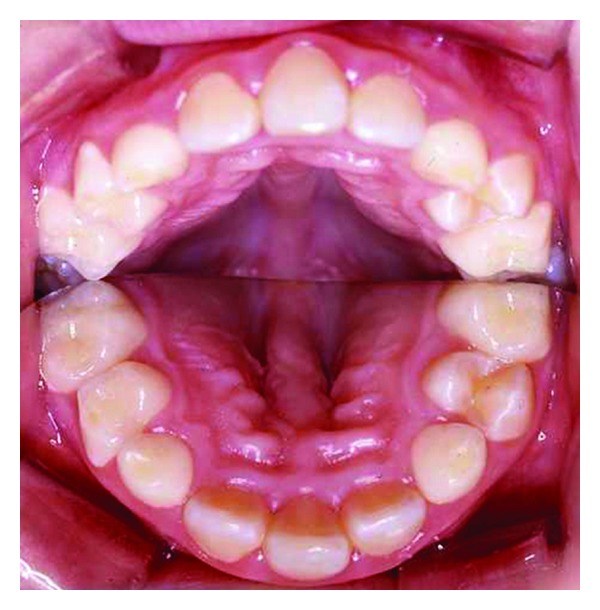
Intraoral photograph with a mirror placed between the dental arches, demonstrating the palate. The figure demonstrates a single central incisor, absence of papilla incisive, and a vault midaxially in the palate. Deviations all occur within the frontonasal field, illustrated schematically in Figure 5. The figure is reprinted with permission from Neuropediatrics 2009;40:280-283 [8].

**Figure 8:**
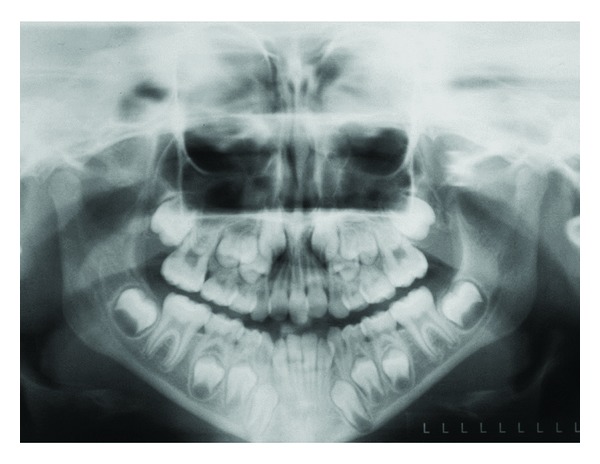
Panoramic radiograph of the dentition in a girl with SMMCI, aged 12 years 1 month, shown in Figure 6. Note the maxillary central incisor, the narrow nasal cavity, and the close-set eyes. Apart from the maxillary front the dentition looks normal. Deviations all occur within the frontonasal field, illustrated schematically in Figure 5.

**Figure 9:**
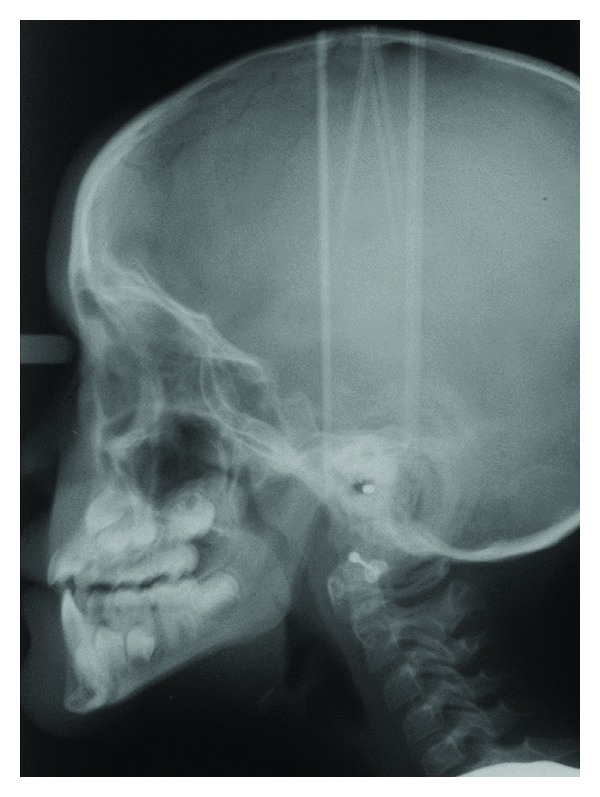
Profile radiograph of girl with SMMCI, aged 12 years 1 month, shown in Figure 6. Note the short anterior cranial fossa, the undeveloped sella turcica, and the maxillary retrognathia. Deviations all occur within the frontonasal field, illustrated schematically in Figure 5.

Recently, also a fusion or nonseparation of the frontal hemispheres has been registered [8]. This is a new finding that expands the craniofacial diagnostics to include brain diagnostics as well. The SMMCI condition is demonstrated by clinical and radiographic images (Figures 6, 7, 8, and 9). The illustrations show that the postnatal findings are in complete concurrence with the prenatal findings stating that only a single field (frontonasal field) is involved in the syndrome.

### 4.2. Several Fields, Exemplified by Trisomy 21

**Figure 10:**
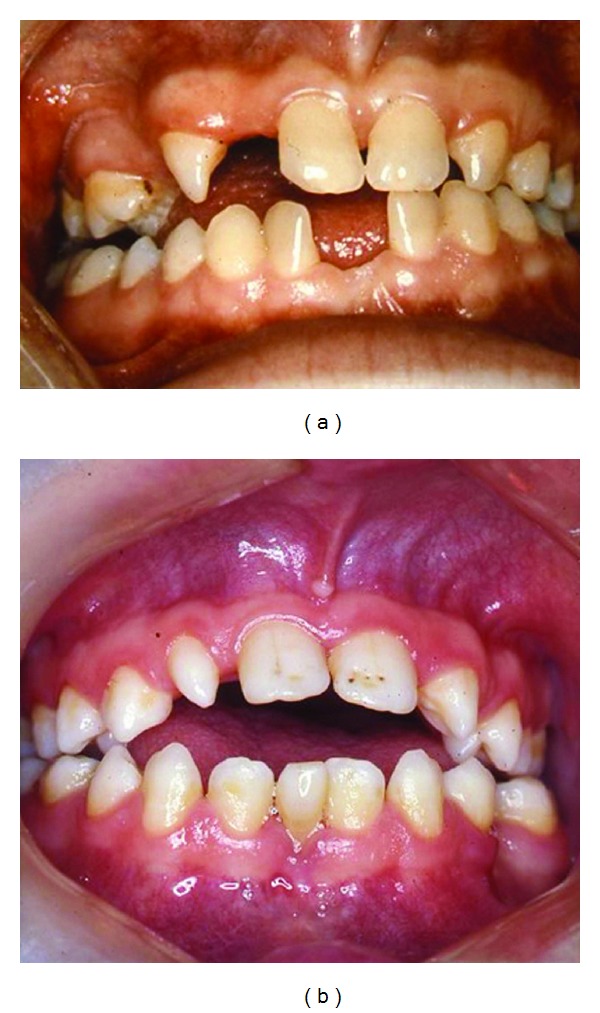
Intraoral photographs of two adult individuals with Trisomy 21/Down syndrome. In the left photograph, agenesis of the maxillary right lateral incisor and premolars is registered. Agenesis is also registered in the mandibular central incisor region. In the right photograph, an anterior vertical open bite is registered. In the maxilla a malformed right lateral incisor, agenesis of the left lateral incisor, and enamel pits at the central incisors are observed. In the mandible, agenesis of one central incisor is observed.

**Figure 11:**
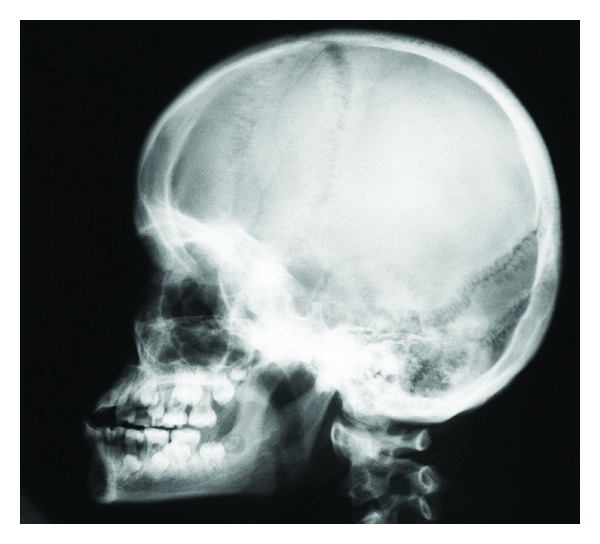
Profile radiograph of a girl with Down syndrome, aged 8 years 6 months. Note the enlarged thickness of the skull, absence of the nasal bone, deviations in the upper contour of the anterior wall of the sella turcica, malformations of the cervical spine, and maxillary retrognathia. Deviations of the skeleton occur in several craniofacial fields.

In several craniofacial syndromes, many of the symptoms cannot be related to fields. One of these syndromes is Trisomy 21/Down syndrome. In Down syndrome, ageneses are registered in all craniofacial fields [70, 71] in locations where ageneses are normally registered [70, 72], that is, lateral incisors in the frontonasal fields; second premolars in the maxillary field; third molars in the palatal field. In the mandible, ageneses are seen most often in the central incisor region, the second premolar region, and third molar region. Compared to normal conditions, the occurrence of agenesis is about 10 times higher in patients with Down syndrome, and especially high in the mandibular incisor region [70]. Also narrow crowns and short roots are observed in the dentition in general [3]. The craniofacial morphology reveals different malocclusions and different malformation signs in the cranium, such as absent or short nasal bone, enlarged thickness of the theka crania, and malformations in the cervical column. The Down condition is demonstrated by clinical and radiographic images (Figures 10 and 11). The illustrations show that the postnatal findings are in complete concurrence with the prenatal findings stating that symptoms occur in several fields in Down syndrome. 

### 4.3. Single Field and Several Fields

This distinction between the location of dental deviations in a single field and in several fields is essential for our insight into craniofacial syndromes. It is believed that the dentition will become central in diagnostics and evaluation of the pathogenesis behind craniofacial syndromes. In a recent paper, Trainor has highlighted the role of neural crest cells in the aetiology and pathogenesis of Treacher Collins syndrome and furthermore for the potential for prevention [27]. We still need to know the genes responsible for neural crest cell migration.

The pathogenesis of SMMCI is associated with the sonic hedgehog gene that is expressed in the midcranial region anterior to the pituitary gland [31, 69].

In Down syndrome/Trisomy 21, a possible gene dosage effect associated with the extra chromosome may influence the phenotype [73]. The extra chromosome may influence the mitotic activity of cells [74].

Future research in craniofacial syndromology should include extended studies on developmental fields in order to establish a more sufficient background for elucidating the pathogenesis. In the frontonasal segment it would be interesting to focus on fields with dental deviations in the maxillary incisor region such as Kallmann syndrome [75], Rieger syndrome [3], KGB syndrome [3, 76], and cleft lip syndrome [77, 78]. In the anterior incisor field in the mandible, it would be interesting to focus on Ellis-van Creveld syndrome described by Gorlin et al. [3]. Also Williams syndrome [79] and Turner syndrome [12] may contribute to new knowledge on the pathogenesis.

It is important to realize that a syndrome has different phenotypic appearances, ranging from mild to severe. It is also obvious that syndromes that are normally confined to one field may have symptoms outside that specific field, as observed in Rieger syndrome [3]. This may be due to different genotypes. There remains still a need for a systematic analysis of the interrelationship between dental deviations, skeletal deviations, associated organ deviations, body growth, and genotypes. 

In recent years, extensive experimental animal studies have focused in particular on the neural crest cell migration in the craniofacial region. These studies are highly important for understanding human development as well [80]. A study such as the previously described study on Velocardiofacial syndrome, also known as Chromosome 22q 11.2 deletion syndrome [62], with widespread symptoms in the same neural crest developmental field, may influence the classification by Spranger et al. from 1982 [1].

Part 3 shows how developmental fields can explore and advance the concept of dental approaches to craniofacial syndromes.

## 5. Discussion

The great advantage of dental analyses compared to all other analyses performed on human tissue is that deviations in the hard tissue persist and remain stable during the developmental course. As dental tissues do not reorganize, they are easy to analyse and use in analyses of fields. Therefore, a dental approach to craniofacial syndromes by analysis of developmental fields contributes to an understanding of the pathogenesis of craniofacial syndromes.

A problem raised by Hennekam in 2007 is which clinical condition can be called a syndrome and which cannot. There are arguments in favour for using aetiology and pathogenesis as the core issue, but Hennekam [81] also states that there are arguments to make the patient's phenotype decide the syndrome definition.

Another problem that should be solved in the craniofacial analysis is the genetics behind the craniofacial fields. Is there a signalling gradient involved in the cranial pattern formation, such as suggested in limb development? [82].

From an embryological and pathological point of view, it can be presumed that the notochord activates the neural crest cells to migration and that different genes are responsible for the different locations of neural crest cells at the neural tube. This adds another aspect to craniofacial development and syndromology and calls for scientific attention in the future.
